# Frailty and Hip Fracture in Portugal: A Retrospective Single-Center Study Highlighting the Urgent Need to Expand Geriatric Care

**DOI:** 10.7759/cureus.104986

**Published:** 2026-03-10

**Authors:** Guilherme Jesus, Rafela Veríssimo, Tiago Fernandes, Mariana Gonçalves, Fátima Silva, Ana Mondragão, Rosélia Lima

**Affiliations:** 1 Internal Medicine, Unidade Local de Saúde Gaia e Espinho, Vila Nova de Gaia, PRT

**Keywords:** fall-risk-increasing drugs, frailty, geriatrics medicine, malnutrition, portugal’s healthcare

## Abstract

Introduction and aim

Proximal femoral fractures in older adults represent a growing public health challenge, associated with significant morbidity, mortality, frailty, and health costs. The study aimed to define the landscape of Portuguese orthogeriatric care and map pathways for future organizational improvement, education, and research; to characterize clinical and epidemiological features, frailty, fall mechanisms, and medication-related risks among older adults admitted with proximal femoral fractures to Portugal’s pioneer orthogeriatrics unit; and to identify system-level gaps requiring national action. The objective was to describe the clinical, functional, cognitive, and pharmacological profile of older adults with proximal femoral fractures treated in this unit and to contextualize these findings within international evidence to support expansion of geriatric and orthogeriatric services nationally.

Methods

A retrospective observational cohort of all patients aged ≥65 years admitted with proximal femoral fracture to the Unidade Local de Saúde de Gaia e Espinho (ULSGE) orthogeriatrics unit between 1 January and 31 December 2023 was analyzed. Comprehensive multidisciplinary geriatric assessment applied the following validated instruments: 4AT, Mini Nutritional Assessment (MNA), and social evaluation. Clinical Frailty Scale (CFS) and Charlson Comorbidity Index (CCI) were also performed. Polypharmacy was defined as ≥5 regular or potentially inappropriate medications (PIM); fall-risk-increasing drugs (FRIDs) were classified using the Beers Criteria and STOPP/START. Fall circumstances were categorized from patient/caregiver/eyewitness reports. Outcomes included in-hospital mortality and discharge destination.

Results

Among 230 admissions, the median age was 85 years (IQR: 80-89); 175 (76%) were women. Frailty was prevalent (140, 61% with CFS ≥5; 96, 42% with CFS 6-8), with substantial multimorbidity (mean CCI: 4.9). Cognitive vulnerability was frequent: 48 (21%) had probable delirium or severe impairment at admission, and 41 (18%) developed in-hospital delirium. Nutritional compromise: 55 (24%) malnourished, 106 (46%) at risk, and all with CFS ≥7 nutritionally compromised. Polypharmacy affected 183 (79.6%, mean: 7.2 drugs); FRID exposure was high (statins 120, 52%; PPIs 102, 44%; benzodiazepines 94, 41%; antipsychotics 61, 26%; loop diuretics 76, 33%). Falls were predominantly modifiable and extrinsic: hazardous home environments (64, 28%); imbalance/gait disturbance (44, 19%); non-adherence or improper use of mobility aids (29, 13%); postural instability (26, 11%). In-hospital mortality was two (0.87%); 150 (66%) were discharged home, 29 (13%) to rehabilitation units, and eight (3%) were newly institutionalized.

Conclusion

This orthogeriatric cohort demonstrates a high burden of frailty, malnutrition, cognitive impairment, and polypharmacy, together with modifiable environmental and medication‑related fall risks. The favorable in-hospital mortality supports the effectiveness of integrated orthogeriatric models compared to traditional wards. National priorities should include formal recognition of geriatrics, scale-up of orthogeriatric services beyond major cities, systematic nutrition and delirium pathways, and structured deprescribing programs targeting FRIDs to improve survival, function, quality of life, and equity of care for Portugal’s rapidly aging population.

## Introduction

Europe is undergoing an unprecedented demographic transformation, with a sustained rise in the population aged 65 years and over. The concomitant increase in age-related morbidity, particularly fragility fractures of the proximal femur, has established hip fracture as a sentinel event in geriatric medicine. This clinical entity represents a critical convergence point for chronic disease burden, frailty, cognitive impairment, malnutrition, and polypharmacy [1]. Portugal, in alignment with its Southern European neighbors, has years of accelerated population aging and a persistent increase in the incidence and complexity of geriatric syndromes. Yet, the organizational and educational infrastructures to meet this demand remain immature - comprehensive geriatric training, structured multidisciplinary teams focused on elderly patients, and orthogeriatric units are underdeveloped. Geriatric medicine itself is not recognized as a medical specialty, but rather only as a post-graduate competence since 2014. This regulatory status continues to constrain workforce development, the expansion of clinical services, and the effective delivery of integrated care [2,3].

The orthogeriatric approach represents an integrated care framework combining geriatric expertise with orthopedic surgical intervention, aimed at delivering comprehensive and holistic treatment for elderly patients sustaining hip fractures [4]. The overwhelming majority of hip fractures (HFs) presentations occur in aged populations, predominantly following fall events that account for more than 95% of cases. Such fractures arise through the synergistic interaction between skeletal fragility and elevated fall propensity [5]. Elderly patients with HF typically present with complex clinical profiles characterized by multiple chronic conditions and geriatric syndrome burden, predisposing them to elevated peri-operative complication rates, suboptimal surgical recovery, substantial mortality risk (approaching 20%), impaired ambulation, and lasting functional limitations affecting approximately half of survivors [6].

Data from European and international cohort studies consistently show that integrated orthogeriatric frameworks yield reduced mortality rates, decreased post-operative morbidity, abbreviated hospitalization durations, and expedited transitions to home-based recovery [7]. Collaborative models that unite traumatology and geriatrics services have proven superior at achieving improved patient outcomes while demonstrating fiscal efficiency [8]. Conversely, across numerous Portuguese hospital settings, elderly HF patients continue to receive care exclusively from orthopedic surgery teams, thereby risking inadequate attention to acute medical complications, pre-existing chronic disease management, systematic fall risk evaluation, and evidence-based osteoporosis intervention.

Despite this, the diffusion of orthogeriatric models in Portugal is gradual and inconsistent, with most hospitals lacking dedicated multidisciplinary teams and services, and services virtually unavailable outside the greater Lisbon and Porto regions. The Orthogeriatric Unit at Unidade Local de Saúde Gaia e Espinho (ULSGE), created in 2015, is Portugal's first dedicated geriatric unit. This pioneering initiative set new national standards for admission criteria, accepting all patients aged 65 years or older with proximal femoral fracture except those who are both bedbound and under active palliative care; notably, non-bedbound palliative patients are included, reflecting a broad and inclusive clinical ethos.

The implementation of collaborative care involving geriatrics-trained internists and orthopedic surgery specialists proved operationally viable, with the interprofessional team comprising physiatrists, physical therapists, nursing professionals specialized in general and rehabilitation care, clinical nutritionists, pharmacists, and social services practitioners [4].

This study provides a detailed analysis of patients admitted in 2023, exploring the interdependent syndromes of frailty, malnutrition, multimorbidity, and polypharmacy; the prevalence and clinical relevance of fall-risk-increasing drugs (FRIDs); and the adverse health outcomes arising from deficiencies in preventive care, delays in specialist assessment, and fragmented medication reviews.

## Materials and methods

A retrospective observational cohort analysis was conducted on all patients aged 65 years and older admitted to the ULSGE orthogeriatric unit with a diagnosis of proximal femoral fracture between January 1 and December 31, 2023. The sole exclusion criterion was active palliative care bedbound status.

A comprehensive assessment at admission was undertaken by the multidisciplinary team using standardized and internationally validated instruments. Demographic data comprised age, sex, and living circumstances (alone, with family/caregivers, or institutionalized). Chronic disease burden was quantified via the Charlson Comorbidity Index (CCI), providing a validated prognostic estimate [9]. Frailty was scored using the Clinical Frailty Scale (CFS), which is a nine‑point clinical scale ranging from 1 (very fit) to 9 (terminally ill); patients were stratified as robust (CFS ≤3), vulnerable (CFS 4), frail (CFS 5), moderately frail (CFS 6), and severely frail (CFS ≥7) according to international conventions [10].

Cognitive profile was evaluated through the 4AT instrument, calibrated for delirium and severe cognitive impairment as follows: scores ≥4 indicated probable delirium or marked cognitive dysfunction [11]. In this orthogeriatric unit, the 4AT is systematically applied at admission before surgery; the present analysis uses admission scores. Repeat assessments during hospitalization are performed as clinically indicated, but were not collected systematically for this study. Nutritional status was determined by the Mini Nutritional Assessment (MNA), classifying patients as well‑nourished (MNA >11), at nutritional risk (MNA 8‑11), or overtly malnourished (MNA ≤7) [12].

Medication regimens at admission and during hospitalization were retrieved from clinical and pharmacy records. Polypharmacy was defined as the regular use of five or more drugs or potentially inappropriate medications at the time of admission, consistent with international standards. Prevalence of FRIDs, including statins, proton pump inhibitors, benzodiazepines, antipsychotics, loop diuretics, selective serotonin reuptake inhibitors (SSRIs), beta‑blockers, and angiotensin‑converting enzyme (ACE) inhibitors/angiotensin receptor blockers (ARBs), was classified using the American Geriatrics Society 2019 Beers Criteria and Euro Screening Tool of Older Persons’ Prescriptions/Screening Tool to Alert to Right Treatment (STOPP/START) v3 [13,14].

Circumstances of the fall were gathered from patient, caregiver, and eyewitness accounts; categories included environmental hazards (rugs, clutter, poor lighting), imbalance, non‑compliance with or improper use of mobility aids, postural instability (orthostatic hypotension, syncope), sensory impairment (visual, auditory), inappropriate footwear or slippery floors, accidents during personal hygiene and cases without any clearly identified cause at admission. Given the multifactorial nature of falls in geriatric populations, each patient’s fall event was analyzed for both primary and secondary contributing circumstances. Consequently, individual patients could be attributed to multiple fall circumstance categories when multiple etiologic factors were identified, resulting in a cumulative frequency count exceeding the total sample size. Similarly, FRID exposure was documented non‑exclusively, as patients commonly received multiple medications classified as fall‑risk‑increasing, with each relevant drug class recorded independently.

Outcomes analyzed included in‑hospital mortality and discharge destination, with data anonymized prior to statistical handling and analysis. Descriptive analysis comprised categorical distributions and means with standard deviations or medians with interquartile ranges, as appropriate; no formal hypothesis testing or multivariable modeling was performed, as the study was designed as an exploratory descriptive cohort, with contextualization against European reference literature and national data repositories.

## Results

A total of 230 consecutive admissions were analyzed. The median age was 85 years (IQR: 80‑89); 175 patients (76.1%) were female and 55 (23.9%) male. At admission, 126 patients (54.8%) were living with family, 55 (23.9%) alone, and 39 (17.0%) in nursing home residential settings; living arrangements were unspecified for 10 patients (4.3%) (Table 1).

**Table 1:** Patient characteristics and demographics.

Characteristics	Value
Total patients, n	230
Age in years, median (IQR)	85 (80-89)
Sex	
Female, n (%)	175 (76.1)
Male, n (%)	55 (23.9)
Living situation	
With family, n (%)	126 (54.8)
Alone, n (%)	55 (23.9)
Nursing home, n (%)	39 (17.0)

High frailty prevalence was evident - 140 patients (60.9%) scored CFS ≥5, including 96 (41.7%) classified as moderately or severely frail (CFS: 6‑8). Only 50 patients (21.7%) with CFS 1‑3 were deemed robust, mostly younger women with preserved functional capacity. The mean Charlson Comorbidity Index was 4.9 (SD: 1.8); 37 patients (16.1%) had CCI <4, while 60 (26.1%) scored >7 (Table 2).

**Table 2:** Clinical assessment scores and syndromes (n=230). CFS: Clinical Frailty Scale; MNA: Mini Nutritional Assessment

Clinical assessment	Value
Clinical Frailty Scale	
Robust (CFS 1-3), n (%)	50 (21.7)
Vulnerable (CFS 4), n (%)	40 (17.4)
Frail (CFS 5), n (%)	44 (19.1)
Moderately frail (CFS 6), n (%)	60 (26.1)
Severely frail (CFS ≥7), n (%)	36 (15.7)
Charlson Comorbidity Index	
Mean (SD)	4.9 (1.8)
CCI <4, n (%)	37 (16.1)
CCI >7, n (%)	60 (26.1)
Cognitive status (4AT)	
Probable delirium at admission (≥4), n (%)	48 (20.9)
Any cognitive impairment/delirium, n (%)	41 (17.8)
Nutritional status (MNA)	
Well-nourished, n (%)	69 (30.0)
At nutritional risk, n (%)	106 (46.1)
Malnourished, n (%)	55 (23.9)

Cognitive impairment and delirium were prevalent geriatric syndromes. According to 4AT, 48 patients (20.9%) were admitted with probable delirium or severe cognitive impairment. During hospitalization, 41 patients (17.8%) developed delirium, typically triggered by acute medical events, infections, medication changes, or, notably, following surgical procedures. Overall, 89 patients (38.7%) experienced some form of delirium or substantial cognitive impairment.

**Figure 1:**
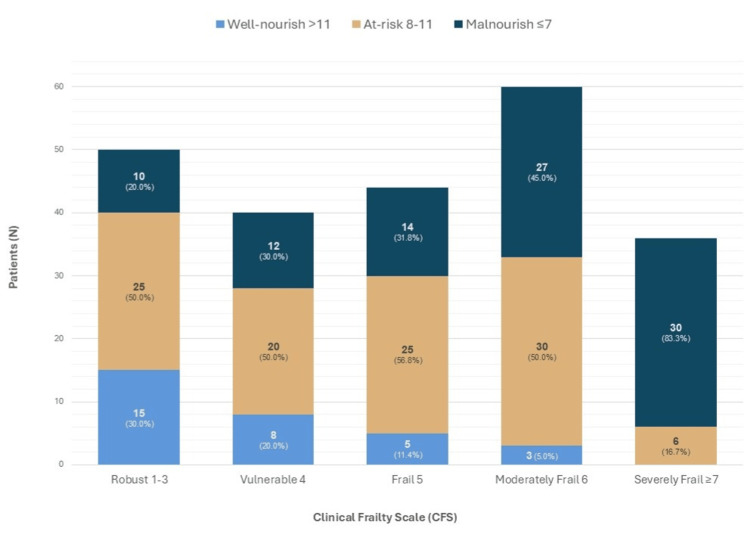
Relationship between frailty (Clinical Frailty Scale) and nutritional status (Mini Nutritional Assessment). Severely frail patients were universally nutritionally compromised, with no patients classified as well-nourished and 83.3% malnourished.

Malnutrition and frailty were tightly linked. MNA scoring revealed that only 69 patients (30.0%) were well-nourished, 106 patients (46.1%) were at nutritional risk, and 55 patients (23.9%) were malnourished. The most vulnerable group, those with CFS ≥7 (n=36), were universally nutritionally compromised (malnourished or at risk) (Figure 1). The nutritionally compromised population also experienced increased rates of delayed recovery, wound complications, and functional decline.

Polypharmacy at admission was nearly ubiquitous - 183 patients (79.6%) were taking five or more medications, with a mean of 7.2 drugs. Analysis of FRID exposure showed high prescription rates - statins in 120 patients (52.2%), proton pump inhibitors in 102 (44.3%), benzodiazepines in 94 (40.9%), atypical antipsychotics in 61 (26.5%), loop diuretics in 76 (33.0%), SSRIs in 62 (27.0%), beta‑blockers in 62 (27.0%) and ACE inhibitors/ARBs in 113 (49.1%). More than three‑quarters of patients were prescribed at least one FRID, and most were exposed to several such medications simultaneously, raising concern for compounding risks.

Scrutiny of fall circumstances revealed multiple concurrent contributing factors in many cases. The frequencies presented reflect all identified primary and secondary circumstances for each patient, which explains why cumulative totals exceed the cohort size (n=230). Similarly, FRID prevalence represents non-exclusive exposure, with most patients prescribed multiple fall-risk medications simultaneously.

Analysis of the fall event showed that modifiable extrinsic factors predominated. Domestic hazards accounted for 64 falls (27.8%), including rugs, clutter, poor lighting, and the absence of handrails. Imbalance and gait disturbance were responsible for 44 falls (19.1%), while non-adherence or improper utilization of mobility aids contributed to 29 falls (12.6%). Postural instability or syncope during transitions to standing was involved in 26 cases (11.3%). Visual impairment accounted for 17 falls (7.4%), inappropriate footwear and slippery surfaces for 13 falls (5.7%), and accidents during bathing or hygiene for 11 falls (4.8%). Notably, 22 falls (9.6%) were attributed to lack of preventive measures, including inadequate safety adaptations in nursing homes (for example, absence of grab bars and poor lighting). Sixteen falls (7.0%) had no clearly identified cause.

Hospital mortality for this cohort was two patients (0.87%). At discharge, 150 patients (65.2%) returned home, 29 patients (12.6%) were transferred to inpatient rehabilitation, 43 patients (18.7%) returned to previous institutional care settings, and eight patients (3.5%) newly required institutional care (Table 3).

**Table 3:** Medication profile and clinical outcomes (n=230). Fall circumstances and FRID categories are non-exclusive; individual patients may contribute to multiple categories, resulting in cumulative frequencies exceeding the total sample size. FRID: fall-risk-increasing drugs; SSRIs: selective serotonin reuptake inhibitors; ARB: angiotensin receptor blocker

Variables	Value
Medication profile	
Polypharmacy (≥5 medications), n (%)	183 (79.6)
Mean number of medications	7.2
Fall-risk-increasing drugs (FRIDs), n (%)	
Statins, n (%)	120 (52.2)
Proton pump inhibitors, n (%)	102 (44.3)
Benzodiazepines, n (%)	94 (40.9)
Atypical antipsychotics, n (%)	61 (26.5)
Loop diuretics, n (%)	76 (33.0)
SSRIs, n (%)	62 (27.0)
Beta-blockers, n (%)	62 (27.0)
ACE inhibitors/ARBs, n (%)	113 (49.1)
Fall circumstances (primary and secondary), n (%)	
Environmental hazards, n (%)	64 (27.8)
Imbalance/gait disturbance, n (%)	44 (19.1)
Non-adherence to mobility aids, n (%)	29 (12.6)
Postural instability/syncope, n (%)	26 (11.3)
Lack of preventive measures	22 (9.6)
Visual impairment, n (%)	17 (7.4)
Inappropriate footwear/surfaces, n (%)	13 (5.7)
Bathing/hygiene accidents, n (%)	11 (4.8)
Unexplained, n (%)	16 (7.0)
Clinical outcomes	
In-hospital mortality, n (%)	2 (0.87)
Discharge destination, n (%)	
Home, n (%)	150 (65.2)
Inpatient rehabilitation, n (%)	29 (12.6)
Previous institutional care, n (%)	43 (18.7)
Newly institutional care, n (%)	8 (3.5)

## Discussion

This study delineates the multidimensional challenges characterizing the Portuguese orthogeriatric population with proximal femoral fractures. Our cohort is defined by a high prevalence of frailty, multimorbidity, malnutrition, and cognitive impairment, further exacerbated by environmental risks and extensive polypharmacy. The demographic distribution, notably the predominance of very old women, aligns with European epidemiological trends for osteoporotic hip fractures [15].

The significant overlap between severe frailty and nutritional compromise reflects a synergistic decline documented in the international literature, in which chronic disease, immobility, and impaired nutritional access reciprocally drive functional deterioration [16]. The high proportion of patients with Clinical Frailty Scale (CFS) ≥5 and elevated CCI scores is consistent with previous series linking these burdens to increased vulnerability to acute stressors and higher predicted one-year mortality [17,18]. Malnutrition serves as a critical prognostic marker for adverse outcomes, including delayed wound healing, infectious complications, and functional loss; it also heightens the risk of recurrent falls and fractures. Despite its clinical impact, systemic preventive care and nutritional interventions remain underdeveloped within the Portuguese healthcare framework [19,20].

Polypharmacy and extensive exposure to FRIDs were pervasive at admission. Despite robust international guidelines advocating for medication review and deprescribing, Portuguese prescribing patterns, particularly concerning statins and proton pump inhibitors, remain among the highest in Europe [21]. The mean medication count and FRID prevalence in our cohort align with, or exceed, data reported in recent European orthogeriatric series [21,22]. This lack of systematic deprescription underscores broader organizational deficits, including suboptimal professional education in geriatric pharmacology and the inconsistent implementation of structured medication reviews [20,21].

This iatrogenic burden significantly compounds the risk of neurocognitive complications. Post-operative delirium is highly prevalent following hip fracture surgery in older adults, representing a multifactorial syndrome with a profound impact on functional recovery and long-term prognosis [11]. In this cohort, cognitive impairment and delirium were identified in nearly 40% of patients, serving not only as direct drivers of morbidity but also as indicators of systemic organizational gaps in care [11,23,24]. Early geriatric assessment, standardized cognitive screening, and protocols designed to minimize psychoactive drug exposure while promoting non-pharmacological prevention remain underdeveloped nationally. Such deficiencies result in missed opportunities for complication risk reduction and the optimization of long-term recovery. Consequently, these findings reinforce the necessity of integrating cognitive screening and delirium prevention bundles into standardized orthogeriatric pathways [23,24].

The pattern of fall etiology reiterates the potential for modification and prevention - most environmental hazards are amenable to intervention, but actual implementation is blocked by socioeconomic constraints, low social support, limited community outreach, and insufficient funding for home adaptation. Non‑adherence to mobility aids, often related to cognitive deficit, lack of patient education, and health‑system inertia, further aggravates risk. These findings parallel European series and reinforce the conclusion that integrated strategies combining medical, social, rehabilitative, and educational approaches are fundamental.

Our in‑hospital mortality of 0.87% lies at the lower end of the commonly cited 4-10% range for European hip fracture admissions under conventional orthopedic management [8,25]. Although direct comparison is limited by differences in case‑mix and follow‑up periods, this result is consistent with international evidence that integrated orthogeriatric care models reduce mortality and improve functional trajectories relative to traditional orthopedic care [4,7,8,25].

The results achieved in the ULSGE unit underscore the clinical efficacy of co-managed care, yet they also expose profound systemic limitations. While our findings align with the superior outcomes reported by established international orthogeriatric services, the majority of Portugal remains devoid of such specialized care models. Only a limited number of units are operational outside major urban centers, and there is still no formal specialty recognition of geriatrics [19,26].

The classification of geriatrics as a mere competence rather than a medical specialty continues to impede the development of structured training pathways and the widespread expansion of services. This regulatory framework hinders the meaningful integration of geriatric principles in both acute and community settings. This challenge is further exacerbated by the fact that geriatric medicine remains an optional subject in undergraduate medical curricula across Portuguese universities. Furthermore, geriatric training in specialty residency programs is facultative rather than mandatory, which limits the proficiency of future specialists in managing the complexities of frailty. As the proportion of the aged population in Portugal continues to increase, the gap between clinical need and service provision will intensify.

The creation and expansion of geriatric units throughout Portugal, extending beyond the orthogeriatric model, must be prioritized at both national and regional levels. Current configurations are excessively hospital-centric and lack integration with community pathways, which undermines functional recovery and the management of preventable complications while compromising health-system efficiency. Furthermore, public health policies in Portugal have historically underinvested in active and healthy aging initiatives. The allocation of per capita resources to gerontological prevention, community support services, and long-term care integration remains significantly lower than benchmarks in Northern and Central Europe [27,28].

Recent Organisation for Economic Co-operation and Development (OECD) data indicate that government expenditure in Portugal on aging-related health and social services represents a markedly smaller share of GDP compared to Scandinavia or the Netherlands. In those regions, comprehensive policies focusing on fall prevention, early community rehabilitation, and age-friendly environments drive superior clinical outcomes [29]. This gap in public investment perpetuates service shortages and limits multidisciplinary community outreach. To align with European standards, it is imperative to expand specialty training through mandatory curricula and residency rotations. These educational reforms must be accompanied by targeted funding for community geriatric programs and policy frameworks that incentivize coordinated care to achieve optimal health outcomes.

Effective care for older adults must be predicated on holistic approaches delivered by multiprofessional teams working through coordinated partnerships. Clinical models should explicitly recognize that aging involves complex interactions between medical conditions, functional capabilities, psychological well-being, and social circumstances. It is therefore recommended that healthcare systems integrate clinical expertise, diagnostic resources, and therapeutic interventions with community support. These elements should be embedded within structured frameworks designed to ensure both the quality and accessibility of care for aging populations.

The imperative for expanding geriatric medical expertise becomes evident when examining workforce projections against demographic realities. Estimates derived from the American Geriatrics Society calculations suggest that Portugal requires approximately 1000 trained geriatricians to meet population-based care standards [30]. This figure represents nearly a 10-fold increase from current capacity, highlighting the magnitude of the workforce deficit facing the country. Without a fundamental restructuring of medical education and training pathways, Portugal will remain unable to address the escalating healthcare demands of its demographic transition. This shortage perpetuates inadequate geriatric care provision and compromises health outcomes for the growing elderly population.

This report is limited by its single-center retrospective design and the absence of systematic longitudinal follow-up post-discharge. Additionally, there was an incomplete recording of certain geriatric assessment tools. Notwithstanding these limitations, the study provides granular and internationally referenced data on a large consecutive cohort, establishing a critical benchmark for future comparisons and strategic planning within the Portuguese healthcare system.

## Conclusions

Portugal faces a critical transition in elder care. This orthogeriatric cohort demonstrates a high burden of frailty, malnutrition, cognitive impairment, and polypharmacy, alongside modifiable environmental and medication-related fall risks. The favorable clinical outcomes achieved in this specialized unit underscore the effectiveness of integrated orthogeriatric models and stand in contrast to the fragmented care prevalent in much of the country. To realign healthcare provision with demographic realities, national priorities must include the formal recognition of geriatrics as a medical specialty and the nationwide expansion of orthogeriatric services beyond major urban centers. Furthermore, the implementation of systematic nutrition and delirium pathways, alongside structured deprescribing programs targeting FRIDs, is essential. Such systemic strategies, encompassing multisector education and integrated community-based programs, are imperative to improve survival, functional recovery, and equity of care for Portugal’s rapidly aging population.
